# Apolipoprotein A-II, a Player in Multiple Processes and Diseases

**DOI:** 10.3390/biomedicines10071578

**Published:** 2022-07-02

**Authors:** Gabriela Florea, Irina Florina Tudorache, Elena Valeria Fuior, Radu Ionita, Madalina Dumitrescu, Ioana Madalina Fenyo, Violeta Georgeta Bivol, Anca Violeta Gafencu

**Affiliations:** Institute of Cellular Biology and Pathology “Nicolae Simionescu” of the Romanian Academy, 050568 Bucharest, Romania; gabriela.florea@icbp.ro (G.F.); irina.tudorache@icbp.ro (I.F.T.); elena.fuior@icbp.ro (E.V.F.); radu.ionita@icbp.ro (R.I.); madalina.dumitrescu@icbp.ro (M.D.); madalina.fenyo@icbp.ro (I.M.F.)

**Keywords:** apolipoprotein A-II, HDL, atherosclerosis, amyloidosis, cardiovascular disease, diabetes, cancer

## Abstract

Apolipoprotein A-II (apoA-II) is the second most abundant apolipoprotein in high-density lipoprotein (HDL) particles, playing an important role in lipid metabolism. Human and murine apoA-II proteins have dissimilar properties, partially because human apoA-II is dimeric whereas the murine homolog is a monomer, suggesting that the role of apoA-II may be quite different in humans and mice. As a component of HDL, apoA-II influences lipid metabolism, being directly or indirectly involved in vascular diseases. Clinical and epidemiological studies resulted in conflicting findings regarding the proatherogenic or atheroprotective role of apoA-II. Human apoA-II deficiency has little influence on lipoprotein levels with no obvious clinical consequences, while murine apoA-II deficiency causes HDL deficit in mice. In humans, an increased plasma apoA-II concentration causes hypertriglyceridemia and lowers HDL levels. This dyslipidemia leads to glucose intolerance, and the ensuing high blood glucose enhances apoA-II transcription, generating a vicious circle that may cause type 2 diabetes (T2D). ApoA-II is also used as a biomarker in various diseases, such as pancreatic cancer. Herein, we provide a review of the most recent findings regarding the roles of apoA-II and its functions in various physiological processes and disease states, such as cardiovascular disease, cancer, amyloidosis, hepatitis, insulin resistance, obesity, and T2D.

## 1. Introduction

High-density lipoprotein (HDL) is an important lipoprotein that is involved in lipid metabolism, playing an essential role in cholesterol efflux [1]. Apolipoprotein AII (apoA-II), a 17.4 kDa protein, is part of HDL particles and contributes up to three dimers per lipoprotein particle, representing as much as 20% of the HDL protein content, second to apoA-I [2]. Due to a higher affinity for lipids as compared with apoA-I, apoA-II appears to have a significant impact not only on HDL properties but also on apoA-I conformation, playing a key role in stabilizing HDL structure [2].

ApoA-II functions have been intensively studied but remain poorly understood since clinical and epidemiological studies issued conflicting results. In contrast to the genetic studies regarding apolipoprotein A-I (apoA-I), those involving apoA-II have not fully clarified its role in atherosclerosis. While human apoA-II deficiency was reported to have little influence on lipoprotein levels and the coronary artery disease risk [3], murine apoA-II deficiency caused HDL deficit in mice [4], suggesting that the effect of apoA-II on HDL is quite different in humans and mice.

Studies in various strains of mice fed with regular chow or atherogenic diets have led to contradictory results. Human apoA-II overexpressed in mice displaced apoA-I from HDL, possibly due to the higher affinity for lipids, and thus impaired postprandial triglyceride-rich catabolism [5,6,7]. Knocking out apoA-II diminished HDL-cholesterol levels and increased the clearance of lipoprotein remnants with atherogenic properties [4]. HDL enriched in ApoA-II, isolated from mice expressing human apoA-II, efficiently protected VLDL from oxidation as compared to the control HDL [8]. However, in another study, the opposite effect was revealed, as dyslipidemic mice overexpressing human apoA-II showed accelerated atherosclerosis and reduced antioxidant HDL activity [9]. The detrimental effects of human apoA-II enrichment were related to its ability to displace other proteins from HDL, such as apoA-I and paraoxonase [5,6,9].

In this review, we summarize the main features of apoA-II and its physiological functions, and we present the involvement of apoA-II in various diseases, such as cardiovascular disease, cancer, amyloidosis, hepatitis, and diabetes.

## 2. ApoA-II Expression and Structural-Functional Properties

### 2.1. ApoA-II Gene

**Figure 1:**
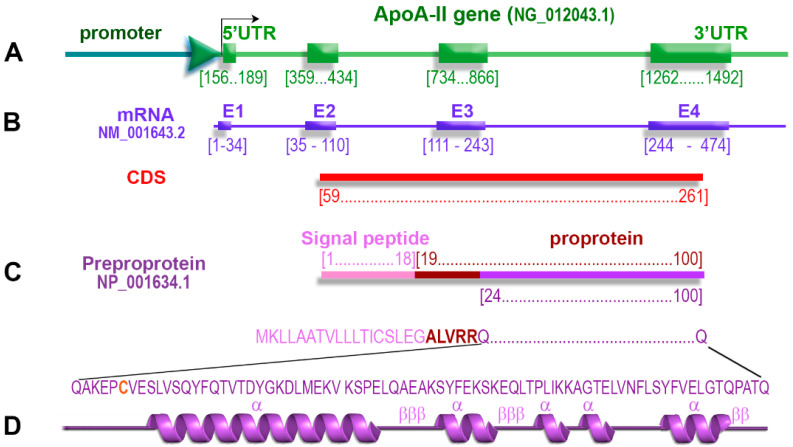
The structure of the human ApoA-II gene. (**A**) Schematic illustration of ApoA-II gene containing the promoter, 5′UTR (untranslated 5′ region), four exons, three introns, and 3′UTR (untranslated 3′ region). (**B**) The mRNA of ApoA-II transcribes all four exons (E1–E4). The coding sequence of the apoA-II gene spans from the second exon to the fourth exon, respectively, from position 59 to 261. (**C**) The preproprotein consists of the signal peptide of 18 amino acids and the proprotein. (**D**) Mature apoA-II protein is formed after the proteolytic cleavage of the ALVRR sequence, and its 77 amino acid sequence is indicated. Human apoA-II has a single cysteine residue (Cys6) involved in the formation of heterodimers with other apolipoproteins. ApoA-II contains amphipathic α-helices in tandem, which are connected through β-turns. Abbreviations: UTR, untranslated region; E, exon; CDS, coding sequence; α, α-helix; β, β-turn.

The ApoA-II gene is a member of the multigenic superfamily of apolipoproteins. In humans, the ApoA-II gene is located on chromosome 1 in region 1q21-q23 [10]. In mice, the ApoA-II gene is located on chromosome 1 close to the *Ath-1* gene, which was associated with the early development of atherosclerotic lesions after a high cholesterol diet [11]. The human ApoA-II gene contains four exons and three introns [12], and its main structural features are depicted schematically in Figure 1.

### 2.2. Regulation of ApoA-II Gene Expression

**Figure 2:**
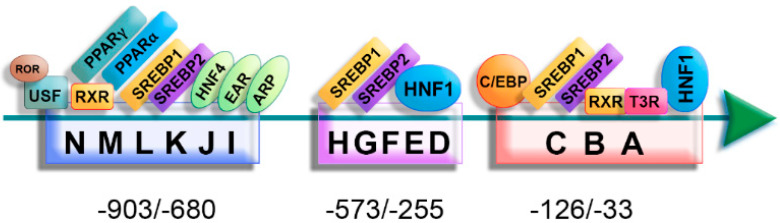
Regulatory elements and transcription factors for *ApoA-II* gene expression control. The regulatory elements within the *ApoA-II* promoter are: (i) proximal (A–C), located in the region -126/-33, (ii) intermediate (D–H), located in the region -573/-255, and (iii) distal (I–N) located in the region -903/-680. *ApoA-II* expression is regulated by many transcription factors and nuclear receptors, such as ROR (Retinoic Acid-Related Orphan Receptor), USF (Upstream Stimulatory Factor), RXR (Retinoid X Receptor), PPAR (Peroxisome Proliferator-Activated Receptors), SREBP (Sterol Regulatory Element-Binding Proteins), HNF (Hepatocyte Nuclear Factor), C/EBP (CCAAT/enhancer-binding protein), and T3R (Thyroid hormone Receptor).

The apoA-II plasma level is influenced by the rate of synthesis rather than by its catabolism [13]. The transcription of the *ApoA-II* gene is mainly controlled by a complex of 14 regulatory elements (A→N) located in the −903/−33 region of the promoter [14], as summarized in Figure 2. Studies of the 5′ deletions and DNase footprinting showed the importance of different fragments of this region in *ApoA-II* gene transcription: the −911/−614 region is essential for transcriptional activation and the −860/−614 region is critical for the hepatic and intestinal transcription; the −853/−671 region was characterized as a liver-specific enhancer element [14,15]. The distal elements (K, L, and N) are essential for the efficient transcription of the *ApoA-II* gene, but not sufficient, since the specific expression of the *ApoA-II* gene in the liver is controlled by the synergistic interaction between transcription factors binding both proximal and distal elements of the *ApoA-II* gene promoter [14].

Zannis’ group revealed that SREBP-1 and SREBP-2 proteins recognize the regulatory elements B-E and K of the promoter, while SREBP-1 also bind on HI elements [16,17], but their role in regulating the *ApoA-II* gene transcription is not entirely elucidated yet. Other transcription factors, such as USF (upstream stimulatory factor) [18], C/EBP (CCC Enhancer-Binding Protein) [19] ARP-1, EAR-2, EAR-3, and HNF-4 [20], also bind the regulatory elements on the *ApoA-II* promoter and modulate its expression (Figure 2).

Metabolites and hormones influence *ApoA-II* transcription, affecting the protein plasma level. Human *ApoA-II* expression is regulated by nuclear receptors, such as PPARα (Peroxisome Proliferator-Activated Receptor α), RXR (Retinoid X Receptor), and RORα (RAR-Related Orphan Receptor α). *ApoA-II* gene promoter is trans-activated by RXR (X-linked retinoid receptor) ligands (such as 9-cis-retinoic acid) through activation of PPAR-γ. Fibrates act on the J element of the *ApoA-II* promoter by activating RXR-PPARα, thus increasing the apoA-II synthesis in humans [21]. Retinoids and thyroid hormones modulate *ApoA-II* gene expression by synergistic or antagonistic interactions between RXRα/T3Rβ heterodimers and the ubiquitous transcription factor USF2a [18].

Interestingly, in both humans and mice, a positive association between blood glucose and plasma apoA-II was revealed, and the binding of HNF4-α to hormone-responsive elements was found responsible for the apoA-II upregulation by glucose [22]. Furthermore, an increased apoA-II concentration causes hypertriglyceridemia and lowers plasma HDL levels; this dyslipidemia induces glucose intolerance and the ensuing high blood glucose enhances the apoA-II transcription, leading to a vicious circle that may cause type 2 diabetes (T2D).

The inflammatory stress affects the expression of apoA-II [23] as well as that of apoA-I [24] and apoE [25,26,27]. However, the regulatory mechanisms target the transcription, following NF-kB binding to the apoA-I and apoE promoters [24,25], while for apoA-II the process is post-transcriptional, and no binding was demonstrated for NF-kB to the apoA-II promoter [23].

### 2.3. The Polymorphisms of the ApoA-II Gene with Clinical Significance

Several polymorphisms of *ApoA-II* have been identified in the gene, but only two of them were confirmed to be pathogenic according to ClinVar (nih.gov, accessed on the 20 June 2022). The main characteristics of these SNPs are summarized in Table 1.

The most important polymorphism of the *ApoA-II* gene is rs5082 (−265T/C), found in the middle of the D element of the gene promoter. Thus, this SNP may affect the binding of the transcription factors on the promoter, modulating the gene expression. Recently, a clinical trial (NCT03452787) showed that saturated fatty acid consumption combined with the −265T > C variants modify the epigenetic status of the regulatory region of the *ApoA-II* gene [28].

Takada and coworkers found that the *ApoA-II* promoter activity of the −265C variant in vitro was reduced to 70% of that of the promoter bearing the predominant −265T nucleotide [29]. The C variant of −265T/C polymorphism was associated with a low plasma level of apoA-II, but also with a small waist circumference, and a high postprandial metabolism of VLDL in healthy men [30]. Thus, compared with TT homozygotes, individuals with CC or CT variants of *ApoA-II* −265T/C polymorphism have a lower level of postprandial hypertriglyceridemia, associated with a decreased risk of cardiovascular disease [31,32]. A positive correlation was also established between the −265T/C polymorphism, dietary fat, and the risk of obesity [33]. Interestingly, in the case of low saturated fat intake (<22 g/day), this SNP was not associated with obesity, but when saturated fat intake was high (≥22 g/day), significant differences in body mass index or obesity were found between C- and T- carriers [34]. Moreover, significantly higher levels of LDL-cholesterol and LDL/HDL in CC carriers were detected, in correlation with the saturated fat intake [35].

The polymorphism rs771259264 was revealed to be the cause of the first case of familial apoA-II deficiency that was reported from Hiroshima, Japan [3]. This polymorphism leads to the splice-junction alteration, blocking the splicing of the third intron and thus preventing the formation of the functional mRNA [3].

### 2.4. ApoA-II Protein

ApoA-II protein was discovered about 50 years ago as the second major HDL protein [36]. ApoA-II protein is a component of the plasma in humans, rodents, simians, and fish, and it is absent or lowly expressed in rabbits, pigs, dogs, and chickens, as reviewed in [37]. Human apoA-II is a glycosylated protein synthesized mainly in the liver and to a lesser extent in the intestine as a pre-pro-apoA-II precursor containing 100 amino acid residues, as reviewed in [38]. Human apoA-II plasma concentration is ~25–40 mg/dl, and its half-life is four days [39]. The levels of apoA-II are inversely associated with coronary artery disease risks [40].

### 2.5. Primary Structure of apoA-II

**Figure 3:**
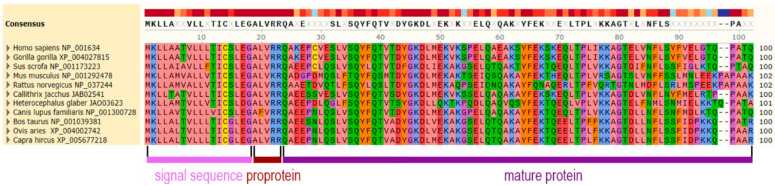
The alignment of apolipoprotein A-II preproprotein from various mammalian species. In the upper part, there is the consensus sequence with a threshold of 75%. The sequences are marked with the color highlighting the physicochemical properties of the amino acids. The alignment was performed using SnapGene—MUSCLE software version 3.8.1551 (GSL Biotech LLC, San Diego, CA, USA).

The protein sequence is well-conserved in mammalians. In Figure 3, the alignment of apoA-II preproprotein sequences from various mammals is shown and the similarities are highlighted in colors related to the physicochemical properties of the amino acids. The primary structure of murine apoA-II differs by ~40% from its human counterpart. In humans, gorillas, and porcine, apoA-II has a single cysteine residue (Cys6) involved in the formation of either homodimers or heterodimers with other apolipoproteins, such as apoE [41] and apoD [42]. The absence of the cysteine residue in murine apoA-II sequence murine determines a monomer structure of the protein [43].

### 2.6. Secondary Structure of apoA-II

**Figure 4:**
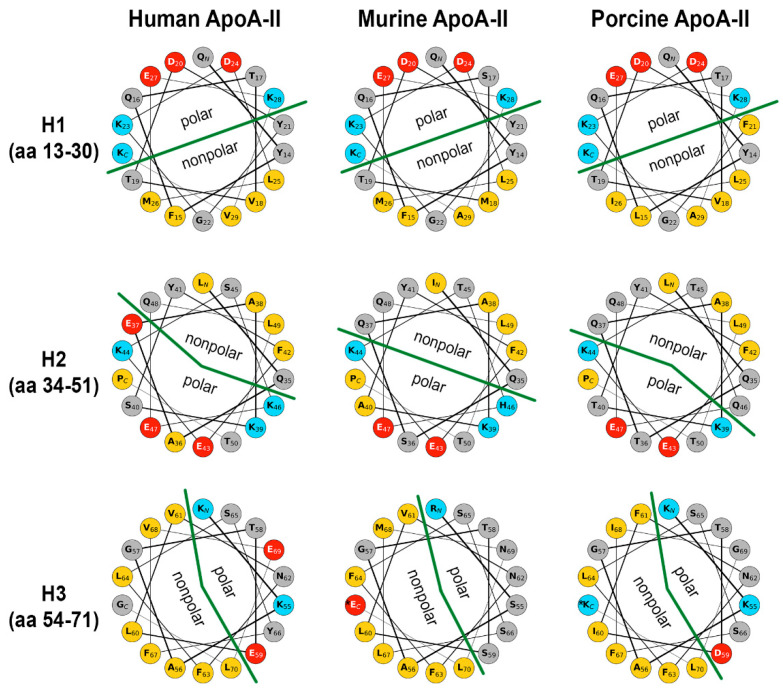
Comparison between the secondary structures of apolipoprotein A-II from human, murine, and porcine origin. The HelicalWheel tool of the Galaxy CPT public platform (https://cpt.tamu.edu/galaxy-pub, accessed on the 20 June 2022) was used to generate the plots. Amino acid (aa) color code: yellow-hydrophobic; grey-polar, uncharged; red-negatively charged; blue-positively charged. The green line demarcates the polar from the non-polar regions in the helical wheels. Note the presence of a charged E residue in the murine nonpolar part of H3, and a K residue in the porcine nonpolar part of the H3 region of apoA-II (marked with *) is introducing a disruption in the non-polar region, as compared with the human homolog. However, since this residue is at the C-terminus of H3, it should not significantly alter the nonpolar character of the face. The sequences used for plots are NP_001634.1 for human apoA-II, NP_001292478.1 for murine, and NP_001173223.1 for porcine apoA-II.

ApoA-II protein contains three amphipathic α-helices in tandem, comprising amino acids 13–29, 34–50, and 54–70, respectively; these helices are connected through two β-turns (amino acids 30–34 and 49–53) [44]. Helical wheels representation of the helix domains of apoA-II of human, murine, and porcine origin is depicted in Figure 4. In the first helix (H1), the mutations in positions 18, 26, and 29 are conservative, and that in position 29 is semi-conservative. Overall, the hydrophobicity of the nonpolar face of H1 is not significantly affected. In the second helix (H2), due to mutations in positions 37 and 46, the nonpolar face may describe a larger angle from human to mouse to pig. The third helix (H3) consists mostly of hydrophobic and polar uncharged residues, thus offering a vast lipophilic area. The most notable difference is the number and/or distribution of the few charged residues, and these may be interesting for further studies in protein–lipid interactions.

Since the third α-helix of apoA-II is more hydrophobic than the first two helices, it endows the C-terminal domain with a higher lipid-binding affinity, as compared to that of the N-terminal domain. A synthetic peptide study showed that the shortest C-terminal domain of apoA-II that can bind phospholipids involves the third α-helix and the second β-turn, which includes the N-terminal part of the second α-helix [44]. By contrast, in apoA-I and apoE apolipoproteins, only the C-terminal domain is required for association with lipids [45,46]. In vitro studies showed that the monomeric form of apoA-II displaces the same amount of apoA-I from HDL as the dimeric form [47]. ApoA-II helices are more hydrophobic and less amphipathic than those formed by apoA-I. Moreover, the N- and C-terminal domains of apoA-II have weaker cooperativity compared to that of other apolipoproteins [44].

### 2.7. Quaternary Structure of apoA-II

Human ApoA-II circulates in plasma as monomers, homodimers, or heterodimers with apoE [41] and apoD [42]. The apoA-II homodimer has two subunits of 77 amino acids, covalently linked by a disulfide bridge through a Cysteine residue at position 6 [48,49]. As seen in Figure 3, gorilla and pig apoA-II also contain cysteine in position 6 and, thus, the molecules can form dimers.

### 2.8. Co-Translational Processing of apoA-II

The co-translational cleavage of the signal peptide from the human pre-pro-apoA-II gives rise to pro-apoA-II, which contains 82 amino acid residues [APOA2—Apolipoprotein A-II—Proteomics (nextprot.org, accessed on the 20 June 2022)]. Upon loading with lipids, apoA-II protein dimerizes and is secreted extracellularly. ApoA-II lipid loading and dimerization occur within two hours after protein synthesis. Lecithin–cholesterol acyltransferase (LCAT) participates in the fusion of LpA-I particles with LpA-II particles and, consequently, LpA-I/A-II particles are formed [50].

### 2.9. Post-Translational Modifications of apoA-II

The post-translational cleavage of pro-apoA-II leads to the mature form of apoA-II, which consists of 77 amino acid residues [APOA2—Apolipoprotein A-II—Proteomics (nextprot.org, accessed on the 20 June 2022)] as a result of proteolytic of the pro-peptide ALVRR from the N-terminus domain. The amino acids arginine, histidine, and tryptophan are missing from the sequence of mature human apoA-II [39]. The post-translational modifications of apoA-II include: (i) O-glycosylation of Thr42 [51], (ii) phosphorylation of Ser 54 and Ser 68 [52], (iii) oxidation of Met 49 [53], and (iv) sialylation [54]. In the culture medium of HepG2 cells, half of the amount of apoA-II was O-glycosylated, and it was reported that O-glycosylation influenced the affinity for lipids [55].

### 2.10. ApoA-II Isoforms

A series of apoA-II isoforms were identified so far, including dimeric and monomeric proteoforms. The isoforms of dimeric apoA-II are identical in the first 74 amino acids, while the amino acids in the carboxyl-terminal position differ [56]. ApoA-II has a major isoform with pI 5 and several minor isoforms that are either more basic or more acidic than the major isoform [39]. The five circulating apoA-II dimeric isoforms are -ATQ/-ATQ (apoA-II-1, 17.380 Da), -ATQ/-AT (apoA-II-2, 17.252 Da), -AT/-AT (apoA-II-3, 17.124 Da), -AT/-A (apoA-II-4, 17.023 Da), and -A/-A (apoA-II-5, 16.922 Da), and they are identified both in healthy individuals and in individuals with various pathologies [56,57,58,59,60] (Table 2). For example, -ATQ/-AT isoform has a low level of expression in patients with autoimmune pancreatitis, chronic pancreatitis, or pancreatic cancer [56,57,59], while -ATQ/-ATQ isoform has an elevated level in patients with autoimmune pancreatitis [56]. In T2D, a high degree of oxidized -AT/-AT and -ATQ/-AT isoforms has been reported [61]. Recently, the monomeric Cys/ATQ, Cys/AT, and Cys/A were identified by top-down proteomics in participants in the CARDIA study [62]. A recent report concluded that there is no significant association of apoA-II isoforms with a risk of myocardial infarction, despite that they found an inverse association between AT/AT isoform and myocardial infarction risk, but it was diminished after adjustment for smoking or drinking only [63].

## 3. Physiological Roles of apoA-II

By interaction with other apolipoproteins and molecules, apoA-II is involved in a series of processes, among which HDL remodeling and cholesterol efflux are the most important.

HDL particles undergo continuous remodeling in plasma. Studies performed with transgenic mice revealed that overexpression of human apoA-II protein led to the formation of smaller and heterogeneous HDL particles compared with HDL particles in the control group [64], while overexpression of murine apoA-II -induced the formation of larger HDL particles than those in the control group [65]. The formation of small HDL particles was associated with low HDL-cholesterol levels [64], and large HDL particles were associated with high HDL-cholesterol concentrations [65]. Certain HDL subfractions might have a significant role in preventing atherosclerosis as compared to others, and modifications of these subfractions may affect the atheroprotective effect of HDL, although the HDL level is not modified or is even increased [66].

Regarding the content in apoA-I and apoA-II, HDL may be classified into two subpopulations, based on the presence of apoA-I and apoA-II: HDL particles containing only apoA-I (known as LpA-I) and HDL particles containing apoA-I and apoA-II (known as LpA-I: A-II); these two HDL subpopulations differ in their structural stability and metabolic fate [67]. The majority of the proteins associated with LpA-I/A-II are involved in lipid transport, whereas those associated with LpA-I are involved in hemostasis, metal ion binding, protease inhibition inflammatory response, and immune response [68]. ApoA-II is bound to HDL particles more stably than apoA-I, and, depending on its concentration, the former may displace apoA-I from HDL particles [69].

Cholesterol efflux from peripheral tissues is the first step in reverse cholesterol transport. Cholesterol efflux can occur by passive diffusion (nonspecifically) or by a specific, energy-dependent mechanism. Contradictory data exist about the efficacy of LpA-I: A-II to promote cholesterol efflux. Many data obtained before the year 2000, suggested that LpA-I is more active in the reverse transport of cholesterol than LpA-I: A-II, because it can induce cholesterol efflux through both the nonspecific and the specific mechanism, as reviewed in [38]. However, more recently, Melchior et al. demonstrated that LpA-I/A-II particles promote a superior level of cholesterol efflux to LpA-I through a mechanism dependent on ABCA1 [68].

Human apoA-II overexpression in C57BL/6 mice revealed pleiotropic effects on HDL structure and function, including increased antioxidant and anti-inflammatory activities, and augmented oxidative phosphorylation in the respiratory chain in white adipose tissue [70].

Mice transiently overexpressing human apoA-II showed improved glucose tolerance, stimulating insulin secretion by pancreatic β-islets [70]. Intriguingly, the findings obtained by transiently overexpressing apoA-II in adult mice differ significantly from data obtained in apoA-II transgenic mice, showing that apoA-II increases body weight gain and reduces glucose tolerance [71]. It is possible that (i) apoA-II expression in the transgenic mice triggers developmental effects on insulin secretion, or (ii) apoA-II induces beta-cell proliferation after adenoviral transduction, as demonstrated in diabetes-resistant B6-ob/ob mice, where adenoviral overexpression of apoA-II contributed to the adaptive islet hyperplasia and, thus, prevented these mice from severe diabetes [72].

## 4. Role of apoA-II in Various Pathologies

Participating in HDL synthesis and remodeling, apoA-II plays an important role in lipid metabolism and associated diseases, such as cardiovascular diseases and metabolic syndrome. However, independent of lipoprotein metabolism, other roles were attributed to apoA-II in immunity, cancer, and amyloidosis.

### 4.1. Role of apoA-II in Atherosclerosis and Cardiovascular Disorders

Atherosclerosis is a chronic disease that affects the medium and large arteries and occurs as a result of the progressive lipid accumulation in the intima of blood vessels, leading to the dysfunction of the vascular endothelium and smooth muscle cells, as well as a chronic state of inflammation [73]. It is well known that low HDL cholesterol is a risk factor for coronary artery disease and elevated HDL cholesterol is associated with a low risk of developing atherosclerosis [74]. In atherosclerosis, there are structural and functional changes in HDL particles [75]. The atheroprotective effect of HDL was explained by the presence of two enzymes that inhibit LDL oxidation in vitro by destroying the biologically active phospholipids from oxidized LDL: paraoxonase [76] and PAF-AH (platelet activator factor-acetyl hydrolase) [77]. Dysfunctional HDL contains lower levels of apoA-I and paraoxonase, losing both antioxidant and anti-inflammatory capacity as well as the ability to promote cholesterol efflux [78]. Mice are deficient in CETP, an enzyme essential for HDL modification; therefore, rabbits are a suitable animal model for the study of HDL because they resemble humans concerning CETP and lipoprotein metabolism. However, normal rabbits genetically lack endogenous apoA-II. In 2009, Koike and coworkers obtained human apoA-II transgenic rabbits (containing a 3-kb human *ApoA-II* genomic fragment) generated by classic transgenesis. The plasma level of human apoA-II in the transgenic rabbits was found to be similar to those of healthy humans (~30 mg/mL); however, these rabbits exhibited increased plasma levels of total cholesterol, triglycerides, and phospholipids, as well as reduced HDL cholesterol levels [79]. The changes in the lipoprotein metabolism of these transgenic rabbits were explained by the inhibition of hepatic lipase by apoA-II [80]. Dyslipidemia described in the human apoA-II transgenic rabbits resembles that of human familial combined hyperlipidemia (FCH), having increased apoA-II levels [79]. Moreover, these transgenic white rabbits expressing human apoA-II genomic DNA were protected against cholesterol-diet-induced atherosclerosis [81]. The anti-atherogenic effects of apoA-II in these rabbits were explained by the fact that apoA-II enrichment of HDL particles enhanced the cholesterol efflux activity and the anti-inflammatory functions of HDL. In 2021, Koike and coworkers obtained rabbits in which endogenous apoA-I was replaced through the knock-in with human apoA-II, using TALEN technology [82]. On a standard diet, these rabbits presented low plasma triglycerides due to enhanced clearance of triglycerides-rich particles and high lipoprotein lipase activity as compared to control rabbits, increased HDL-cholesterol levels, and HDL particles enriched in apoE, apoA-IV, and apoA-V [82]. Interestingly, when fed a cholesterol-rich diet for four months, apoA-II knock-in rabbits developed fewer atherosclerotic lesions, as compared to wild-type rabbits since they were resistant to diet-induced hypertriglyceridemia [82].

In contrast to the protective role of human apoA-II demonstrated in rabbits, apoA-II studies using transgenic mice produced different results. Overexpressed human apoA-II increased the atherosclerosis susceptibility of these transgenic animals more than the murine protein [74].

Transgenic mice overexpressing murine apoA-II (containing ~4.5 kb of sequence 5′ to the first exon and ~8.5 kb of sequence 3′ to the fourth exon of the mouse *ApoA-II* gene) had a reduced level of paraoxonase, which decreased the HDL atheroprotective effect and exhibited hypertriglyceridemia, obesity, and atherosclerosis, characteristics associated with the insulin resistance syndrome [66,71,83,84]. Although the transgenic mice with murine apoA-II overexpression had high levels of HDL, their atherosclerotic lesions had a bigger area than those of the control mice, even after a low-fat chow diet [66,84]. This result might be explained by the fact that apoA-II replaces apoA-I [69] and apoE [84] from HDL particles and these particles with high apoA-II content are not fully functional [85].

Transgenic mice overexpressing human apoA-I and apoA-II proteins became prone to develop atherosclerosis as opposed to mice that overexpressed only human apoA-I protein [74]. Overexpression of human apoA-II was correlated with a decrease in HDL-cholesterol levels [86]. Human apoA-II overexpression in transgenic mice was associated with atherosclerosis, but also with increased triglyceride levels and obesity [74]. Human apoA-II excess contributes to postprandial hypertriglyceridemia by inhibiting lipolysis of triglyceride-rich lipoproteins, regulating lipoprotein lipase at least in part by apoE, apoC-II, and apoC-III displacement from the surface of HDL particles [7]. The effects of human apoA-II on HDL metabolism were inhibition of LCAT and CETP and activation of liver lipase [49]. Familial combined hyperlipidemia (FCH) subjects exhibit high apoA-II levels, hypercholesterolemia, increased body fat, insulin resistance, altered free fatty acid metabolism, as well as a high rate of coronary artery disease [87]. Transgenic mice overexpressing human apoA-II resemble this human disorder, and, thus, these mice are useful for investigating this disease.

Clinical studies revealed interesting results. A study performed on a human subject cohort showed that apoA-II is inversely associated with the risk of future coronary artery disease [40,88]. Clinical trials in patients whose primary lipid abnormality is a low HDL-cholesterol level revealed a reduced incidence of coronary artery disease events after therapy using gemfibrozil, an agonist of PPARα that increases the expression of numerous genes involved in lipid metabolism, including apoA-II and apoA-I [89,90].

The CARDIA (Coronary Artery Risk Development in Young Adults) study published recently indicated proteoform—specific associations between apoAI and AII and the cardiometabolic parameters. The proteomic analysis revealed that the dimer of singly truncated chains AT/AT of apoA-II had the strongest association with HDL-cholesterol and HDL efflux. Moreover, ATQ/Cys and ATQ/ATQ clustered together, with significant positive correlations with obesity markers. Overall, while most proteoforms showed no significant associations with phenotype, the clustering pattern indicated that proteoform-to-phenotype association is dependent both on the number of truncations and the number of polypeptide chains [62].

The ratio of apoA-II/apoB was identified as a better prognostic parameter for lipoprotein-associated risk of postoperative mortality, as compared with the established lipid-risk parameters in patients with high-grade carotid stenosis; therefore, apoA-II/apoB ratio was proposed as an additional risk-prediction tool [91]. Although structural and functional differences between HDL particles with and without apoA-II (LpA-I: A-II and LpA-I) have not been fully distinguished, LpA-I particles are considered to be more responsible for the anti-atherogenic properties of HDL and LpA-I: A-II particles represents rather an atherogenic indicator [92].

### 4.2. Role of apoA-II in Metabolic Syndrome

Alterations in the levels of apoA-II and other apolipoproteins (apoC-II, apoC-III, and apoE) were associated with metabolic syndrome [93]. The involvement of apoA-II in the regulation of free fatty acids and cholesterol metabolism may play a role in the promotion of insulin resistance [49].

Overexpression of murine apoA-II protein in transgenic mice increased insulin levels and decreased hydrolysis of triglycerides in adipose tissue, leading to their accumulation and insulin resistance; moreover, fatty acid oxidation decreased in skeletal muscle, resulting in triglyceride accumulation, insulin resistance, and obesity [66].

Interestingly, the diabetes-related locus includes the region where the *ApoA-II* gene is located (1q21–q24) [94]. T2D is associated with abnormal lipid metabolism and an increased level of oxidative stress [95]. T2D patients have HDL particles with a high content of oxidized fatty acids and high levels of lipid peroxidation products. The oxidative stress in diabetes occurs as a consequence of free radical overproduction that results from glucose self-oxidation [71]. In T2D patients, the apoA-II protein with a high oxidation level of methionine residues and with a limited cholesterol efflux capacity has been identified [61]. The oxidation of methionine residues in apoA-II protein increases the hydrophilicity of the molecule and reduces its affinity for lipids, having a role in disrupting lipid metabolism [53]. Some apoA-II isoforms were detected in T2D patients as the result of various truncations at the C-terminus and/or oxidation of a Met26 (Table 2). The ratio of oxidized apoA-II monomers and oxidized apoA-II isoforms were found to be higher in T2D patients than in healthy subjects [61]. Recently, it was revealed that the half time of apoA-II protein is decreased in patients with diet-controlled T2D (51.9 ± 17.3 h), as compared to the healthy volunteers (91.9 ± 23.1 h) [96]. Moreover, apoA-II polymorphisms were found to be associated with obesity and insulin resistance. The −265T/C *ApoA-II* polymorphism was found in familial combined hyperlipidemia patients [30,97,98]. The CC homozygotes were associated with body mass index and waist circumference larger than compared with TC heterozygotes or TT homozygotes [97,99]. Although not recognized as a pathogenic SNP yet, the polymorphism −492T/C was associated with obesity (CC homozygotes had higher obesity risk than carriers of the T variants) [97].

### 4.3. Role of apoA-II in Immunity

The biological role of apoA-II extends well beyond its function in lipid transport and metabolism. For instance, apoA-II downregulated the oxidative burst and the production of cytokines by human neutrophils in a similar manner to apoA-I [100]. Interestingly, human apoA-II increased human monocyte responses to lipopolysaccharide (LPS) by suppressing the activity of LPS-binding protein [101]. Apolipoproteins (such as apoE, apoA-I, and apoA-II) modulate the response to LPS by direct binding to LPS or by modifying the functions of LPS-responsive cells [102]. It is well established that during infection and inflammation there are significant changes in lipoprotein metabolism, as comprehensively reviewed by the group of Grunfeld [103]. Wait et al. monitored the concentration of serum acute-phase proteins in apolipoprotein A-I and A-II transgenic mice for 4 days after the LPS challenge, and they identified 28 distinct proteins by HPLC MS [104]. Interestingly, Bagdade et al. characterized the changes of lipoprotein subclasses during endotoxemia in human volunteers and identified the formation of new LpA-II:B:C:D:E particles as new acute phase lipoproteins with immunoregulatory properties [105].

Administration of apoA-II suppressed Concanavalin A-induced hepatitis in apoA-II deficient mice [106]. When the immunosuppressive mechanism of apoA-II was investigated, it was revealed that this disorder is suppressed by apoA-II via reduction of the production of IFNγ by CD4 T cells. Therefore, apoA-II administration in combination with glucocorticoids was suggested as a therapy for autoimmune hepatitis.

A few recent reports involving apoA-II emerged in the context of the COVID-19 pandemic. An abrupt decrease in serum cholesterol levels was reported in patients after the onset of COVID-19 disease [107]. In particular, the decrease in HDL-cholesterol levels positively correlated with the severity of COVID-19 [108]. Furthermore, elevated levels of serum HDL-cholesterol were correlated with protection against hospitalization for COVID-19 [109]. As would be expected from changes in the levels of serum HDL-cholesterol, the levels of most of the apolipoproteins associated with HDL were also decreased in COVID-19, including apoA-I and apoA-II [110]. Recently, it was demonstrated that native HDL isolated from human plasma exhibited in vitro antiviral activity against SARS-CoV-2 and after glycation, its antiviral activity was impaired [111]. Thus, apoA-I and apoA-II might have an impact on the severity of SARS-CoV-2 infection, but further research is needed to clarify their antiviral activity [112].

### 4.4. Role of apoA-II in the Early Detection of Cancer

ApoA-II isoforms and abnormal apoA-II expression levels were correlated with various malignancies.

ApoA-II isoforms are considered candidate biomarkers for the early detection of pancreatic cancer. ApoA-II isoform levels in the serum of patients with pancreatic cancer are higher as compared to healthy individuals [57,59]. The patients with autoimmune pancreatitis have a high level of the apoA-II isoform -ATQ/-ATQ and a low level of isoforms -AT/-AT [56] and -ATQ/-AT in plasma [56,57]. In addition, the plasma level of the isoform -ATQ/-AT was found to be reduced in patients with pancreatic cancer [57] or with autoimmune pancreatitis (Table 2). To quantify the plasma concentrations of apoA-II-ATQ and apoA-II-AT isoforms, a novel sandwich ELISA was established [113]. In 2007, Ehmann et al. identified an apoA-II isoform in which a C-terminal glutamine residue is missing in the apoA-II homodimer considering it a potential cancer biomarker [114]. In 2010, Xue et collaborators reported a substantial reduction of apoA-II isoforms in patients with pancreatic adenocarcinoma [115]. Two years later, two differently altered plasma apoA-II dimers were associated with pancreatic cancer [56]. Thus, serum analysis of altered apoA-II isoforms was proposed by several independent groups as an additional serum biomarker for the early detection of pancreatic cancer.

Altered apoA-II expression was specifically associated with prostate cancer, pancreatic cancer, hepatocellular carcinoma, gastric cancer, and myeloma, as reviewed in [116]. As a prostate cancer marker, apoA-II was found to be overexpressed in patients with prostate cancer and prostate-specific antigen < 4 ng, but with no clinical symptoms [117]. By contrast, patients with pancreatic cancer had lower serum apoA-II levels than healthy individuals [57]. It is worth mentioning that Scavenger Receptor B1 (SR-B1) expression is increased in pancreatic cancer tissues, resulting in high uptake of HDL by cancer cells, consequently explaining the reduced levels of apoA-II detected in pancreatic cancer tissue [118]. 

### 4.5. Role of apoA-II in Amyloidosis

Amyloidosis represents a group of hereditary systemic diseases caused by the accumulation of misfolded, undegradable proteins [119]. In these pathologies, soluble proteins change their native conformation and generate β-folded cross-linked structures, leading to the formation and storage of insoluble fibrils. Apolipoproteins such as apoE3, apoA-I, and apoA-II play an important role in the pathology of amyloidosis. It was revealed that apoA-II amyloidosis is due to the T→G replacement in the TGA STOP codon of the *ApoA-II* gene, resulting in a variant of apoA-II with a 21-residue peptide extension on the C-terminus [120]. Mutations of STOP to serine, arginine, or leucine were found to be associated with apoA-II amyloidosis [121,122,123]. The substitution generates a new restriction site for *Bst*N1 that is useful to identify individuals with this apoA-II alteration. However, the amyloidosis caused by apoA-II mutations is rare, as apoA-II is protected in vivo from misfolding [124] due to the structural properties of helices 1 and 3 which are inserted deeply into the lipid layer [123]. In vitro, in the absence of lipids, apoA-II is prone to incorrect folding, forming β-structures.

To study the pathogenesis and identify a potential treatment for apoA-II amyloidosis, a transgenic mouse model was generated using the DNA of the human amyloid-associated *ApoA-II* gene [125,126]. Accumulation of amyloid fibrils was detected in the liver, kidney, spleen as well as cardiac tissue [125,126]. Murine apoA-II amyloidosis was also studied by other researchers using intravenous apoA-II fibril injections and they detected amyloid accumulation after 4 months as compared to 2 months after fibril injection [127,128]. Korenaga et al. described heavy apoA-II amyloid accumulation in C57BL/6 and BDF1 mice, isolated amyloid fibrils, and characterized them at morphological and biochemical levels [129]. Severe amyloidosis developed in mice in which apoA-II proteins had glutamine at position 5 because this residue was involved in polar interactions that stabilized the β-sheet conformation. Interestingly, another group of researchers revealed that the endoplasmic reticulum stress is induced by apoA-II amyloid accumulation in the kidney and the liver, but not in the cardiac tissues [127]. Thus, in mice with apoA-II amyloidosis, an increased expression of heat shock protein A5 (HSPA5), as well as numerous endoplasmic reticulum stress-related proteins, including activating transcription factor 4 and 6 (ATF4 and ATF6), eukaryotic translation initiation factor 2 alpha kinase 3 (EIF2AK3), X-box-binding protein 1 splicing (XBP1S), DNA-damage inducible transcript 3 (DDIT3), and autophagy protein 5 (ATG5) were found [127]. In addition, the researchers assessed the ratio of Bax/Bcl2 and TUNEL-positive cells and found increased apoptosis in the apoA-II amyloid deposition in the liver and kidney [127]. In C57BL/6J/apoA-I deficient mice, the redistribution of apoA-II in HDL and larger HDL particles increased apoE plasma levels, reduced levels of triglycerides, and HDL-cholesterol, and ensued severe apoA-II amyloidosis [130]. To investigate the role of apoA-II in amyloidosis, apoA-II deficient mice were employed, and it was determined that amyloid deposition was reduced in apoA-II^−/−^ mice, as compared to wild-type mice [131]. Since previous studies showed that N- and C- terminal parts of the apoA-II protein are crucial for amyloid fibrils polymerization [132], an inhibitory model was proposed to prevent fibrils extension. Thus, it was demonstrated in vitro and in vivo that the C-terminal sequence of apoA-II type F (which contains several substitutions) represents a good inhibitor of amyloid fibrils polymerization [133,134]. Recent studies showed that oxidative stress inhibitors (such as tempol and apocynin) reduced apoA-II amyloid deposition [132]. Contrary to expectations, via activation of the PPARα pathway, curcumin increased apoA-II amyloid deposition in mice that were induced to develop systemic amyloidosis [135].

A recent study reported reduced levels of apoA-II proteins (but not apoA-I) in patients with Alzheimer’s disease or mild cognitive impairment, as compared to normal cognitive individuals [136]. In addition, increased apoA-II was found in patients with endocrine-metabolic diseases, as compared to non-endocrine-metabolic diseases individuals, while no difference was found in apoA-II when patients with cardiovascular disease were compared with non-cardiovascular disease individuals [136].

In human apoA-II amyloidosis, drastic renal damage has been observed, and the only valid treatment available so far is dialysis followed by kidney transplantation in the advanced stages of the disease [124]. Therefore, understanding the cause and designing possible therapeutic procedures for this disorder is of utmost importance. Although inherited systemic apoA-II amyloidosis is considered to be a rare disorder in humans, researchers consider that this illness is misdiagnosed [137] since it is confused with the acquired monoclonal immunoglobulin light-chain amyloidosis, and in this case, the illness is not properly treated.

## 5. Conclusions

The major role of apoA-II is connected with lipid metabolism and associated cardiovascular diseases since it is the second most abundant protein in HDL particles. However, its role in HDL functions and metabolism needs to be better clarified due to the controversial literature data. Recent mass spectrometry proteomics data pointed to the importance of subtle modifications of apoA-II associated with different phenotypes. Consequently, it would be highly desirable for future studies to characterize the particular isoforms of apoA-II rather than their bulk amount. Dyslipidemia described in human apoA-II transgenic rabbits resembles the human familial-combined hyperlipidemia since increased apoA-II levels are associated with this disorder. The association of apoA-II with other diseases beyond those related to lipid metabolism was identified. Thus, some modifications of the protein were found to make this protein a precursor of amyloid fibrils. The studies performed on apoA-II amyloidosis using various mice models are valuable for understanding the cause and possible therapeutic procedures for this illness. However, further investigation of the mechanisms by which apoA-II induces amyloidosis is needed.

Last but not least, apoA-II was also considered a cancer biomarker for the early diagnosis of various cancers. In particular, the association of apoA-II alterations with pancreatic cancer is valuable since the detection of early-stage pancreatic cancer of non-symptomatic patients by harmless strategies is imperative to reduce the mortality due to this devastating pancreatic carcinoma.

Despite the numerous biochemical and genetic studies performed so far, more conclusive data are expected in the future to better define the multiple roles of apoA-II in human health and disease.

## Figures and Tables

**Table 1:** Main SNPs of apolipoprotein A-II with confirmed clinical significance.

SNP ID	Clinical Significance and Condition	Chromosome Position	Variation	Location	References
**rs5082**	Pathogenic SNP: Reduced plasma LDL cholesterol Familial hypercholesterolemia 1	161,223,893(-)	G/A	REGULATORY	[28,29,30,31,32,33,34,35]
**rs771259264**	Pathogenic SNP: ApoA-II deficiency, familial (Hiroshima)	161,222,917(-)	C/T	SPLICE_ DONOR	[3]

**Table 2:** ApoA-II isoforms and the pathologies associated with these isoforms.

Isoform	Associated Pathology	Effect of Amino Acid Change	Reference
*-ATQ/-ATQ*	Autoimmune pancreatitis	↑ protein expression	[56]
	Seemingly healthy subjects		[59]
	Autoimmune pancreatitis	↓ protein expression	[56]
	T2D	↑ degree of oxidation	[61]
	Seemingly healthy subjects		[59]
*-ATQ/-AT*	Autoimmune pancreatitis chronic pancreatitis; pancreatic cancer	↓ protein expression	[59]
	T2D	↑ degree of oxidation	[61]
	Seemingly healthy subjects		[56,57,59]
*-AT/-A*	Pancreatic cancer		[59]
*-A/-A*	Pancreatic cancer		[59]

## Data Availability

Not applicable.

## Abbreviations

ABCA1: ATP-binding cassette transporter A1; ApoA-I: Apolipoprotein A-I; ApoA-II: Apolipoprotein A-II; FCH: familial combined hyperlipidemia; HDL: high-density lipoprotein; LDL: low-density lipoprotein; LCAT: Lecithin–cholesterol acyltransferase; LPL: lipoprotein lipase; TG: triglyceride; VLDL: very low-density lipoprotein; LPA: Lipoprotein(a); LpA-I, apoA-I only HDL particles; LpA-I: A-II, ApoA-I and ApoA-II containing HDL particles; LPL: lipoprotein lipase; LPS: Lipopolysaccharide; CETP, cholesteryl transfer protein ester; HNF: hepatic nuclear factor; RXR: Retinoid X Receptor; ROR: RAR-Related Orphan Receptor; SREBP-2: Sterol Regulatory Element Binding Protein-2; C/EBP, CCAAT/CCC: Enhancer-Binding Protein; DBP: D-site Binding Protein; SNP: single nucleotide polymorphism; T2D: type 2 diabetes; USF: upstream stimulatory factor.
